# Analysis of the Relationship between Turning Signal Detection and Motorcycle Driver’s Characteristics on Urban Roads; A Case Study

**DOI:** 10.3390/s19081802

**Published:** 2019-04-15

**Authors:** Alfonso Micucci, Luca Mantecchini, Maurizio Sangermano

**Affiliations:** Department of Civil, Chemical, Environmental and Materials Engineering—DICAM, University of Bologna, viale del Risorgimento 2, 40136 Bologna, Italy; luca.mantecchini@unibo.it (L.M.); maurizio.sangermano@studio.unibo.it (M.S.)

**Keywords:** motorcycle-car crash risk, intersection, vehicle turning signals, logit model

## Abstract

The investigations on the effectiveness of the turn signal in motorcyclists understanding of motorists’ potential intentions in potentially dangerous car–motorcycle interactions and on the relationships among some variables that could influence the perception of rear and front turn signal status are examined in this paper. The investigations have been based on data pooled from the answers of a survey of 136 motorcycle riders, with special regards to the correct detection of turning indicators. Experimental videos have been realized during in-situ simulations, both in urban and suburban areas, recording vehicular interactions in three-leg road intersections, able to potentially generate crash risks, through a 360-camera mounted on a motorcyclist’s helmet. The blinkers detection rate has been combined with other factors related to motorcyclist’s characteristics and test context (e.g., age, gender, location of the test site, presence of a car behind tester vehicles and if the motorcyclist are also habitual car or bicycle drivers) in a stepwise logistic regression that modelled the odds of detecting the turn signal turned on as a function of significant factors. Within the limits of the proposed methodology, the results highlight the low percentage of correct sighting of the turn indicators and confirm the existence of a relation between the detection of the turn indicators aspect and some of the variables considered (e.g., age, being habitual cyclist or car driver and the presence of a car occluding the views), suggesting the opportunity to further investigate the phenomenon through the use of ad-hoc simulations, in order to highlight connections among the factors that can influence the perception of turning indicators in potentially dangerous contexts for cars and motorcycles.

## 1. Introduction

Almost half of all fatalities of road accidents in the world involve users with the lowest level of protection (i.e., motorcyclists, pedestrians, and cyclists). In particular, motorcyclists represent 10% of road deaths in Europe, 20% in America, and 34% in Asian states [1].

In Europe, according to stats [2,3], eleven motorbike drivers or passengers are killed per 100,000 registered motorcycles. More than double compared with just five per same quantity of registered cars. The share of motorcycle riders’ fatalities among all road deaths differs throughout the Europe states, from 5% in the Balkan states to 35% in Greece. From 2011 to 2016 the amount of accidents occurrence and motorcyclists killed or injured in Italy is slightly diminished, with the number of deaths contracting from 205,747 to 175,792 [4]. From that year, the number of fatalities has kept on following a descending pattern.

Other than the higher demise rate, motorcyclists are more likely to get injured when involved in an accident [5]. Horswill and Helman [6] dug into 403 injury accidents in the UK in which either a motorbike or car was part of a head-on impact with a vehicle. About 97% of motorbike riders were hurt or killed in these accidents compared with 51% of car drivers. Since there is a higher likelihood that motorcycle-car collisions can occur at higher velocities than the ones between two cars, they analyzed a sample of 111 motorcycle-car head-on accidents, chosen from the previous dataset, and found out that the riders involved had a 96% chance to get injured compared to just the 1% of car drivers. This big difference seems to confirm that motorcyclists are much more vulnerable than car drivers. At present, we say that regarding the at-risk level, a kilometer travelled by motorbike is equivalent to thirty kilometers travelled by car.

### 1.1. Literature on Motorcycles Crash 

A typical claim of vehicle drivers involved in accidents is that they did not detect motorbikes and their riders in time or did not notice them by any means. Hancock et al. [7] and Wertheim [8] defined two leading factors causing the drivers failing to see bikers: Sensory conspicuity (i.e., motorcycles have a small size) and cognitive conspicuity (i.e., motorbikes are less frequent and hence less expected than other modes of transport). Motorcycles are smaller than cars or trucks, then it is much harder to detect them, also because there is the possibility that they may be hidden behind or above other vehicles. Furthermore, individuals detect objects considering their color, size, shape and kinematic parameters. From far away, motorcycles remind pedestrians or bicycles, aside from their velocity. We should consider that the approach speed of motorcycles is very variable and therefore, more difficult to predict. 

As for motorcyclists, the problem is substantially different: Rarely someone says that they have not seen the car, while many motorcyclists claim to have seen the car they were about to overtake or cross, but did not understand that the vehicle was going to turn because the turn signal was off, while motorists say the turn signal was on.

Undoubtedly, aggressive driving could be deemed as an additional cause to the occurrence of motorcycle crashes [9,10]. This in not only related to the attitude of drivers but also to the fact that, occasionally, they could be more prone to accept risk (e.g., alcohol assumption [10]). Horswill and Helman [6] investigated the motorcyclists’ driving approach and found that they were used to travel at higher speed than cars, overtook more and drove through the small gaps between the other vehicles during rush hours.

Demographic factors play a big role in the risk-taking behavior of motorcyclists. For instance, various investigations [11,12,13,14,15] found that younger bikers have higher probabilities of getting involved in a road accident. Most young male riders drive more dangerously than women and older road users, and have lower likelihoods of detecting hazards [16]. Furthermore, it appears that bikers who see an imminent high-risk event do not embrace precaution actions in order to avoid it, even if they have already experienced a similar situation [17,18].

As indicated by few investigations [19,20,21], there are some distinctive characteristics of motorcycles that make the motorcyclists increasingly inclined to injuries and crashes. First of all, motorcycles usually have higher power-to-weight ratio than cars at a much lower price, making them very affordable for almost everyone. Likewise, being a one-track vehicle, the instability of equilibrium is much more likely to happen compared to cars, especially while braking or on slippery road surfaces. Longitudinal irregularities or cutting of the road surface, and raised zebra crossings, may be the cause to instability condition for the bikes. Further causes of crashes could be the incorrect use of brakes. One crucial representative of a motorcycle is to be a balanced machine. Thus, the improper actuation of the brakes may lead to overturning and skidding conditions. An investigation by Sheppard et al. [22] studied the use of motorcycle brakes by observing the bikers’ behavior at road intersections; in ordinary conditions, more than 30% of riders used just the rear brake while just 10% utilized only the front brake [23].

### 1.2. Literature on Turn Signals Perception

Even though vehicle turn signals advanced and progressed toward become standardized over the course of the last century, many differences emerged between U.S. and European guidelines. The specific distinction between the two guidelines is that Europe requires tail turn signals to be solely yellow, while in the U.S., they can be either yellow or red.

Hence, in Europe, all tail turn signals emit a yellow light, while in the U.S., they can be colored either amber or red. The reason for this choice lies behind the decision made by the “Group de Travail-Bruxelles” (GTB) at an event in 1960. The GTB prescribed the use of amber turn signal to the Europeans and the Americans. In the meantime, United States makers dismissed the exclusive use of amber turn signals in favor of red turn lights, basing their decision on doubtful money saving advantages [24].

In general terms, the exclusive use of amber rear turn signal conditions would be preferred to the situation in which either a yellow or red light can be used. That is because of the perception of chromatic thresholds: a yellow turn signal would be more effectively recognized from other reddish colored turn lights by the difference in color with brake lights for example [25]. In addition, current light technology requires the utilization of discrete light compartments for different colored lamps, guaranteeing that amber turn lights are spatially separated by red brake lights [26]. Lamp separation helps to better distinguish the contrast between the on and off state of the light.

Consistency of guidelines and construction standards throughout the globe is the key aspect to increasing the probability of easily and correctly detecting the turn signal light [27,28]. Two studies have investigated the potential of two different colored turn lights. Luoma et al. [29] noticed that the response time to a braking signal is shorter in the case of a yellow turn signal compared to a red one. In any case, it must be considered that the study was performed outside the U.S., where the presence of both the amber and the red turn signal could have given mixed results [30,31,32]. Braitman et al. [33] pointed out another aspect contributing to crashes and related to lights perception in most people: age. In fact, the number of accidents increases when older people drive, usually at the intersection while turning left.

More generally, there are few studies in the literature that focused on the perception of turn signals by motorcycle riders. Some of these studies compare the hazard perception abilities of motorcyclists and car drivers [6], others investigate the effects of driving experience and confidence on risk perception [34]. Recently, some studies have been performed by using motorcycle driving simulators [35], confirming the effects of latent factors in hazard perception.

## 2. Materials and Methods

**Figure 1:**
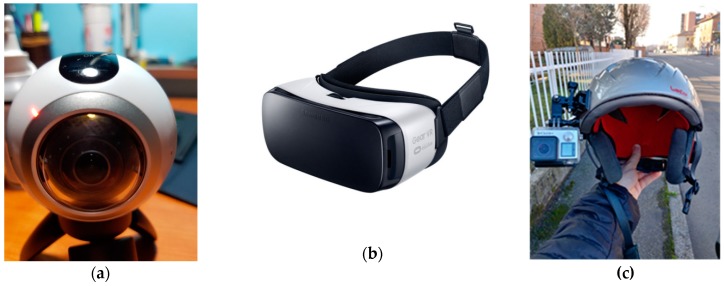
Samsung gear 360 camera (**a**); Samsung Gear VR produced by Oculus (**b**) and GoPro Hero 4 attached to a helmet (**c**).

As already mentioned, the research question that underlies this study is to assess the effectiveness of the turn signal in motorcyclists’ understanding of motorists’ intentions in a potentially dangerous car–motorcycle interaction and to determine whether there are any variables that influence the perception of rear and front turn signal status. To try to answer these questions, some study tests have been designed and performed, recording videos shot by a helmet’s camera. In the survey phase, a virtual reality visor has been used to allow individuals of a selected sample to watch two videos and, finally, respond to a questionnaire. The tools used throughout the study were (see Figure 1):

- Samsung gear 360 camera;
- Samsung gear VR visor;
- GoPro hero 3;
- Samsung galaxy s7 G-930F;
- Action Director Software.

The 360 camera can capture 360-degree videos and photos; it has been attached on a motorcyclist’s helmet and controlled via smartphone. The Samsung Gear VR powered by Oculus (i.e., Gear VR) is a head mounted, virtual reality device which allows the interviewed people to watch the videos realized during the tests in an immersive and realistic way. Finally, the GoPro Hero is the camera that was mounted on the side of the head of the car driver to record, for future insights, the turning signal of the motorcycle approaching the car. On the front panel, a f/2.8 six-element aspherical lens that is supposed to reduce the amount of barrel distortion is installed.

### 2.1. In-Situ Simulations

The simulations have been realized in urban (first three simulations) and suburban (last three simulations) environments because the suburban road environment is usually characterized by a more heterogeneous traffic composition, higher average speeds and by a different driving approach; some cases reported in the literature [14] hypothesize that sub-urban areas have higher hazard levels compared to urban ones.

**Figure 2:**
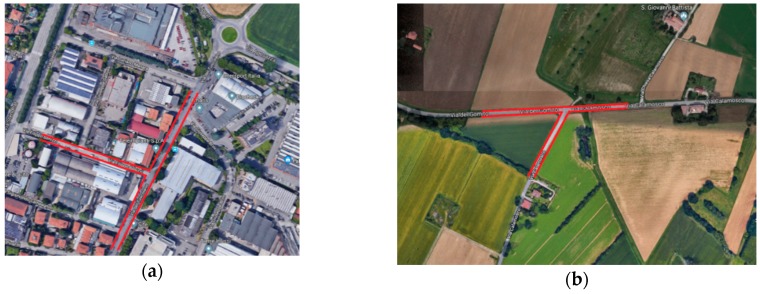
Intersections where the simulations took place, located in urban area (**a**) and in suburban area (**b**). (Coordinates 44°32′07.0″ N, 11°21′33.6″ E for urban intersection and 44°31′38.2″ N, 11°23′38.0″ E for suburban intersection).

The simulations foreseen the use of three vehicles: a motorbike and two sedan cars. The motorbike was identified as “Vehicle A”. The cars, placed steady at the intersection of the two roads (see Figure 2), were identified as “Vehicle B” and “C”, respectively. The vehicle C assumed the role of vehicle occluding the view: it partially prevents the ability of the arriving motorcyclist to see the blinker of the vehicle B during the turning maneuvers. The first set of videos were shot through the camera attached on the motorcyclist helmet (vehicle A); contemporarily, videos inside the car stopped at the intersection were shot using the GoPro Hero mounted on the driver’s helmet.

#### 2.1.1. Simulation 1 (Urban Intersection)

**Figure 3:**
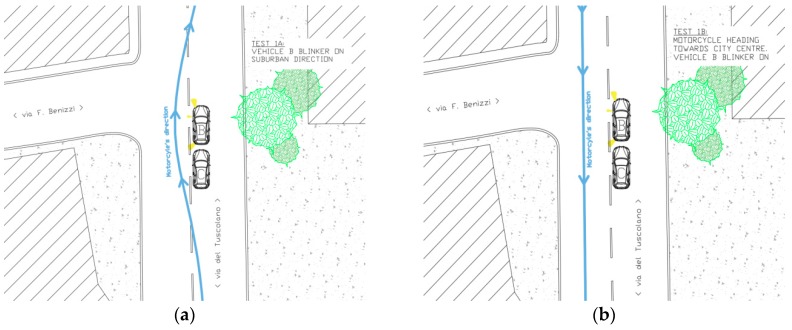
Simulation 1 (**a**) overtaking, and 1 (**b**) going through, schemes.

The first simulation comprised two phases, a and b (see Figure 3):

(a) The vehicle B was stationary at an intersection, in preselection position, with the turning signal on ON. The vehicle C was stationary at intersection, behind the vehicle B, with the turning signal on OFF. The motorcyclist (vehicle A) drove (~50 km/h) along the main road heading from city center towards suburbs area and during the overtaking of the two vehicles B and C the blinker of the motorcycle was turned ON.
(b) The motorcyclist, after the roundabout was entirely travelled, honked the horn to report the beginning of the second part of the simulation. The motorcycle (vehicle A) drove (~50 km/h) along the main road heading from suburbs areas towards the city center. The vehicle B had the turning signal on ON. The vehicle C is in the same position as the simulation 1a.

#### 2.1.2. Simulation 2 (Urban Intersection)

**Figure 4:**
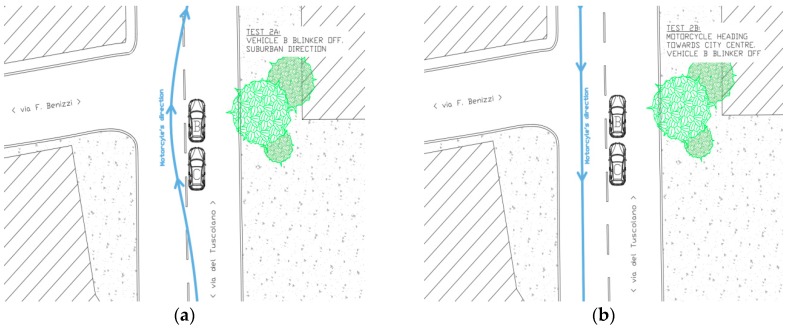
Simulation 2 (**a**) overtaking, and 2 (**b**) going through, schemes.

The second simulation also included two phases, a and b (see Figure 4):

(a) The vehicle B was stationary at an intersection, in preselection position, with the turning signal on OFF. The vehicle C was stationary at intersection, behind the vehicle B, with the turning signal on OFF. The motorcycle (vehicle A) drove (~50 km/h) along the main road heading from city center towards suburbs area and during the overtaking of the two vehicles B and C the blinker of the bike was turned OFF.
(b) The motorcyclist, after the roundabout has been entirely travelled, honked the horn to signalize the beginning of the second part of the simulation. The motorcyclist (vehicle A) drove (~50 km/h) along the main road heading from suburbs areas towards city center. The vehicle B had the turning signal on OFF. The vehicle C was in the same position as the simulation 2a.

#### 2.1.3. Simulation 3 (Urban Intersection)

**Figure 5:**
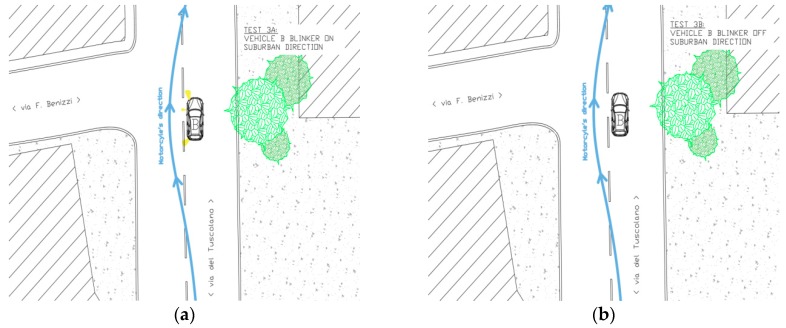
Simulation 3 (**a**) overtaking, and 3 (**b**) overtaking, schemes.

The third simulation was characterized by the fact that the vehicle C was not used. There were two phases, both in suburbs direction (see Figure 5):

(a) The vehicle B was stationary at an intersection, in preselection position, with the turning signal on ON. The motorcyclist (vehicle A) drove (~50 km/h) along the main road heading from city center towards suburbs area and during the overtaking of the vehicle B the blinker of the motorcycle was turned ON.
(b) The vehicle B was stationary at an intersection with the turning signal on OFF. The motorcyclist (vehicle A) drove (~50 km/h) along the main road heading from city center towards suburbs area and during the overtaking of the vehicle B the blinker of the bike was turned OFF.

#### 2.1.4. Simulation 4 (Suburban Intersection)

**Figure 6:**
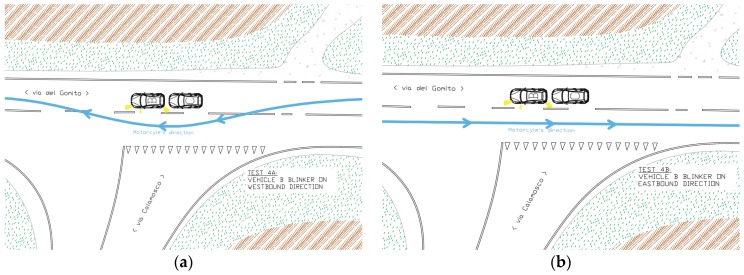
Simulation 4 (**a**) overtaking, and 4 (**b**) going through, schemes.

The forth simulation was comprised of two phases, a and b (see Figure 6):

(a) The vehicle B was stationary at an intersection, in preselection position, with the turning signal on ON. The vehicle C was stationary at intersection, behind the vehicle B, with the turning signal on OFF. The motorcyclist (vehicle A) drove (~50 km/h) along the main road heading westbound and during the overtaking of the two vehicles B and C the blinker of the motorcycle was turned ON.
(b) The motorcyclist, after a U turn, honked the horn to report the beginning of the second part of the simulation. The motorcyclist (vehicle A) drove (~50 km/h) along the main road heading eastbound direction. The vehicle B had the turning signal on ON. The vehicle C is in the same position as in the simulation 4a.

#### 2.1.5. Simulation 5 (Suburban Intersection)

**Figure 7:**
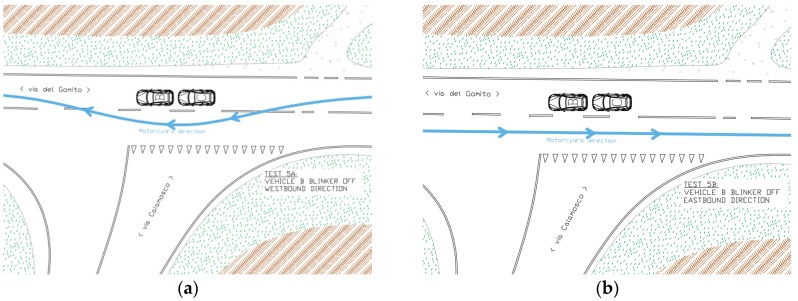
Simulation 5 (**a**) overtaking, and 5 (**b**) going through, schemes.

The fifth simulation was, again, comprised of phases a and b (see Figure 7):

(a) The vehicle B was stationary at intersection, in preselection position. with the turning signal on OFF. The vehicle C was stationary at intersection, behind the vehicle B, with the turning signal on OFF. The motorcyclist (vehicle A) drove (~50 km/h) along the main road heading westbound and during the overtaking of the two vehicles B and C the blinker of the motorcycle bike is OFF.
(b) The motorcyclist, after a U turn, honked the horn to signalize the beginning of the second part of the simulation. The motorcyclist (vehicle A) drove (~50 km/h) along the main road heading eastbound. The vehicle B had the turning signal OFF. The vehicle C was in the same position as in the simulation 2a.

### 2.2. Survey Design, Test Campaign and Data Collection

**Figure 8:**
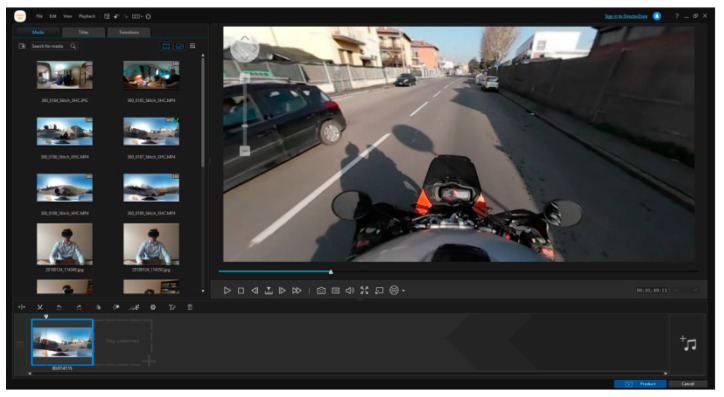
Video editing procedure.

The videos collected from the 360-camera during the simulations have been edited and rendered in the ten videos representing the base for the tests. In order to do so, the editing software “action director” designed and developed by Samsung has been used. For each simulation, the images were treated with cutting and editing procedures (Figure 8), obtaining the final ten videos to be used for the survey phase.

In the producing phase the resolution of 2880 × 1440 pixel and 24 frames per seconds has been selected. This is because the smartphone’s display resolution is the same of the video (and the refresh rate too).

During the survey phase, each participant, belonging to a sample of 136 motorcyclists (the characteristics of which will be illustrated in par. 3.1) watched two of the previously edited videos (representing all the relevant combination of test configuration parameters: the simulation “a” and “b” scheme, the rural or urban environment, the blinkers turned on or off and the presence or not of a vehicle occluding the view) using the Samsung Gear VR, and immediately after viewing the videos they were ask to answer to a questionnaire specifically designed, giving information regarding the status of the turning signal, collecting at the same time other relevant variables (age, gender, being habitual car or bike driver). In particular, as the analysis is referred to a sample of motorcyclists, the knowledge of some latent driving attitudes, as being habitual cyclists or car drivers, is useful to highlight the effects of driving confidence factors.

It is important to note that, during this test campaign, no other parameters related to the behavior of the user sample were detected, such as the reaction time in case of positive detection of the indicator, given that the purpose of the analysis it is only to investigate on the effectiveness of the turn signal and to identify possible relationships between the correct detection of its status and the variables taken into consideration, not to measure other psychophysical or driving-related parameters, the nature of which requires, as known from the literature, different research approaches (use of simulators or direct field tests).

### 2.3. Data Analysis: Logistic Regression Model

Regression methods have gradually become a fundamental aspect of data analysis related to the relationship between a response variable (e.g., occurrence of road accidents) and one or more independent variables (i.e., influencing factors). In the literature, it is widely recognized [36,37] that the utilization of conventional regression analysis is unsuitable to identify probabilistic issues concerning the occurrence of road accidents and to investigate the relationship among crash rate and factors that can influence it. Since the end of the last century, logistic regression has been widely applied to develop accident models and to study crash outcomes [38,39,40].

In statistics, logistic regression or logit regression is a type of probabilistic model. The key mathematical idea that underlies logistic regression is the logit, the natural logarithm of an odds ratio. Often, logistic regression is utilized referring to the problem in which the dependent variable is binary (there are two available categories and problems with more than two groups are known as multinomial logistic regression). It is also used to expect a binary response from a discrete or binary predictor [41]. The probabilities describing the possible outcomes of a single test are modelled, as a function of the attribute’s variables, using a logistic function. A least square test is used to show how good the logistic regression model fits the dataset.

More in detail, a binary output variable Y is given, and the conditional probability Pr (Y = 1|X = x) = E(Y/X) is exhibited as a function of x (*p*(*x*)); any unknown parameter in the function can be estimated by the concept of maximum likelihood. The easiest modification of ln *p*(*x*) is the logistic transformation (i.e., logit), $ln\frac{p}{1-p}$.

Formally, the logistic regression model is:

(1) $$ln\frac{p\left(x\right)}{1-p\left(x\right)}=\beta_{0}+x\cdot \beta$$

Solving for *p*(*x*) gives:

(2) $$p\left(x\right)=\frac{e^{\beta_{0}+x\cdot \beta }}{1+e^{-\left(\beta_{0}+x\cdot \beta \right)}}$$

As logistic regression give as output probabilities, rather than just classes, we can fit it using likelihood. For each component of the dataset, we have a vector of attributes, *x_i_*, and an observed class, *y_i_*. The occurrence of that class is either p(x), if *y_i_* = 1, or 1−p(x), if $y_{i}\;=\;0$. The likelihood function is given by:

(3) $$\ell \left(\beta_{o},\beta \right)=\prod_{i=1}^{n}p{\left(x_{i}\right)}^{y_{i}}{\left(1-p\left(x_{i}\right)\right)}^{1-y_{i}}$$

Typically, to find the maximum probability values, and so to find the unknown values of the parameters that maximize the probability to observe the values given by the model, it is necessary to differentiate the log probability with respect to the parameters, and solve the derivates for zeros. To do that, take the derivative of (3) with respect to one element of β, say $\beta_{j}$.

(4) $$\frac{\partial \ell }{\partial \beta_{j}}=-\sum_{i=1}^{n}\frac{1}{1+e^{\beta_{o}+x_{i}\cdot \beta }}e^{\beta_{o}+x_{i}\cdot \beta }\cdot x_{ij}+\sum_{i=1}^{n}y_{i}x_{ij}=\sum_{i=1}^{n}(y_{i}-p(x_{i};\beta_{o},\beta ))\cdot x_{ij}$$

Equation (4) cannot been solved exactly, usually it can be treated with numerical methods.

## 3. Results

### 3.1. Description of the Variables

One hundred and thirty-six participants took part in the test campaign. They were all motorcyclists and students or members of the staff of the University of Bologna, School of Engineering and Architecture. All participants have been informed about the study purposes and the treatment of personal data. In Table 1 the characteristics of the sample and the variables considered in the analysis are reported. In particular, the sample is uniform concerning gender (43% male), being habitual bicycle drivers (almost 50%). The tests have been performed balancing the presence of the car occluding the view and the urban or sub-urban environment.

The sample is mainly composed by habitual car drivers (68%) and, finally, regarding age, most of the sample was of those under the age of 50 (74%).

### 3.2. Model Estimation

As reported in Section 2.3 a logistic regression model has been specified and calibrated, basing on the results of the performed survey. Table 2 shows the results from fitting all the explanatory variables simultaneously. From the Wald statistic values, it appears that the variables CYC (bicycle driver), CAR (car driver), AGE (age) and OCCL (occluding car) show significant effect at 5% level.

Hence, the logistic regression model calibrated in this study is:

$$p\left(x\right)=\frac{e^{-3.320-1.049\cdot CYC+1.203\cdot CAR+2.171\cdot AGE+0.747\cdot OCCL}}{1+e^{-3.320-1.049\cdot CYC+1.203\cdot CAR+2.171\cdot AGE+0.747\cdot OCCL}}$$

where *p*(*x*) is the conditional probability to correctly detect the car turning indicator in the tested context. The logit of the logistic regression model is given by:

$$ln\frac{p\left(x\right)}{1-p\left(x\right)}=-3.320-1.049\cdot CYC+1.203\cdot CAR+2.171\cdot AGE+0.747\cdot OCCL$$

The fit of the model has been tested considering the log-likelihood statistics:

$$2\left(LL_{1}-LL_{0}\right)=\left(-2LL_{0}\right)-\left(-2LL_{1}\right)\sim \chi^{2}$$

where *LL*_1_ is the log-likelihood of the full model, and *LL*_0_ is the log-likelihood of the reduced model (in particular, the model running with only the intercept). The degrees of freedom (*df*) of the chi-square variable are the number of variables considered in the full model minus the number of parameters in the reduced model. The computed values for the estimated model are:

*LL*_1_ = −78.70

*LL*_0_ = −91.39

*p*-value < 0.001 → the model is significant.

## 4. Model Interpretation and Discussion

First of all, it is important to highlight (Table 1) that only in 40% of the cases, the aspect of the turning indicators has been correctly identified. Moreover, both main effects and interactions among parameters and the conditional probability to detect the turning indicator can be evaluated considering the model coefficients *β_k_*, which directly allows to calculate odds ratio involved in turning signal detection. The odds of a given event are generally defined as the probability of the event occurring divided by the probability of the event not occurring. More in detail, the interpretation of the actual effect of a coefficient in a logistic regression model and its magnitude depends on the possibility to explain and interpret the difference between two logits. The exponent of this difference gives again the odds ratio, which is defined more in detail as the ratio of the odds that the variable examined will be cause to the odds that it will not be cause. All the estimated odds ratios are reported in Table 3, and in the following sub-paragraphs, a brief description of the effects of significant parameters is given.

### 4.1. Impact of Being Habitual Cyclist on Turning Signal Detection

The estimated coefficient of the parameter CYC, according to Table 2, is −1.049; to interpret the effect of this parameter the logit difference can be evaluated as follows:

$$\begin{array}{c} \mathrm{Logit}\;(\mathrm{signal}\;\mathrm{detection}/\mathrm{habitual}\;\mathrm{cyclist})\;=\;-3.320-1.049\cdot CYC+1.203\cdot CAR+2.171\cdot AGE+0.747\cdot OCCL \end{array}$$

$$\begin{array}{c} \mathrm{Logit}\;(\mathrm{signal}\;\mathrm{detection}/\mathrm{non}-\mathrm{habitual}\;\mathrm{cyclist})\;=\;-3.320+1.203\cdot CAR+2.171\cdot AGE+0.747\cdot OCCL \end{array}$$

Logit difference = −1.049·CYC

The odds ratio is: $OddsR=e^{-1.049}=0.35$, meaning that being habitual cyclist seems to reduce the probability of detecting the turning signal by a factor of 0.35.

### 4.2. Impact of Being a Habitual Car Driver on Turning Signal Detection

The estimated coefficient of the parameter CAR, according to Table 2, is 1.203; the logit difference in this case is:

$$\begin{array}{c} \mathrm{Logit}\;(\mathrm{signal}\;\mathrm{detection}/\mathrm{habitual}\;\mathrm{car}\;\mathrm{driver})\;=\;-3.320-1.049\cdot CYC+1.203\cdot CAR+2.171\cdot AGE+0.747\cdot OCCL \end{array}$$

$$\begin{array}{c} \mathrm{Logit}\;(\mathrm{signal}\;\mathrm{detection}/\mathrm{non}-\mathrm{habitual}\;\mathrm{car}\;\mathrm{driver})\;=\;-3.320-1.049\cdot CYC+2.171\cdot AGE+0.747\cdot OCCL \end{array}$$

Logit difference = 1.203·CAR

The odds ratio is, finally: $OddsR=e^{1.203}=3.33$ meaning that the odds of detecting the turning signals being a habitual car driver are 3.33 times higher than those for non–habitual car drivers.

### 4.3. Impact of Age on Turning Signal Detection

The estimated coefficient of the parameter AGE, according to Table 2, is 2.171; this variable has to be treated carefully, because, as previously described, the age of the people in the sample is not uniformly distributed. The association between driver’s age and involvement in road accidents is well known and investigated in the literature for both four wheels (see for example [42]) and two-wheeler vehicles [43]. In particular, it has been shown that most younger motorcyclists have a higher propensity for risky behaviors and these behaviors have been shown to be associated with increased risks of accidents [18]. At the same time, it is widely recognized that many elderly drivers are at particular risks for vehicle crashes in challenging driving environments; this is mainly due to age-related visual, cognitive and physical dysfunctions [33,44].

The logit difference, evaluated as follows, confirms these last considerations:

$$\begin{array}{c} \mathrm{Logit}\;(\mathrm{signal}\;\mathrm{detection}/\mathrm{age}\;<\;50)\;=\;-3.320-1.049\cdot CYC+1.203\cdot CAR+2.171\cdot AGE+0.747\cdot OCCL \end{array}$$

$$\begin{array}{c} \mathrm{Logit}\;(\mathrm{signal}\;\mathrm{detection}/\mathrm{age}\;\geq \;50)\;=\;-3.320-1.049\cdot CYC+1.203\cdot CAR+0.747\cdot OCCL \end{array}$$

Logit difference = 8.77·AGE

The odds ratio is, finally: $OddsR=e^{2.171}=8.77$ meaning that the odds of detecting the turning signals for drivers with age < 50 are 8.77 times higher than those for older drivers.

### 4.4. Impact of Presence of An Occluding Car on Turning Signal Detection

The estimated coefficient of the parameter OCCL, according to Table 2, is 0.747; to interpret the effect of this parameter the logit difference can be evaluated as follows:

$$\begin{array}{c} \mathrm{Logit}\;(\mathrm{sign}\;\mathrm{detect}/\mathrm{presence}\;\mathrm{of}\;\mathrm{occluding}\;\mathrm{car})\;=\;-3.320-1.049\cdot CYC+1.203\cdot CAR+2.171\cdot AGE+0.747\cdot OCCL \end{array}$$

$$\begin{array}{c} \mathrm{Logit}\;(\mathrm{sign}\;\mathrm{detect}/\mathrm{absence}\;\mathrm{of}\;\mathrm{occluding}\;\mathrm{car})\;=\;-3.320-1.049\cdot CYC+1.203\cdot CAR+2.171\cdot AGE \end{array}$$

Logit difference = 0.747·OCCL

The odds ratio is, finally: $OddsR=e^{0.747}=2.11$ meaning that the odds of detecting the turning signal in the presence of an occluding car are 2.11 times higher than the situation in which the car is not present.

## 5. Conclusions

In this study, we evaluated the effectiveness of cars’ turn signals (blinkers) and explored the possible determinants of the correct detection of their status by motorcyclists in urban and sub-urban road environments. The analyses have been performed realizing some videos during specific simulations, both in urban and sub-urban areas, using a 360-camera attached to a motorcyclist’s helmet. The simulations have been set to reproduce vehicular maneuvers (i.e., left turn in three-leg road intersections) able to potentially generate dangerous situations. The videos have been edited and rendered, realizing the base for a test campaign followed by a survey consisting of a questionnaire specifically designed and subjected to a sample of 136 motorcyclists. The detection or not of the blinkers (turning indicators) by users has been combined with other factors (e.g., age, gender, location of the test site, presence of a car occluding the view, motorcyclist also habitual car or bicycle driver) in a stepwise logistic regression that modelled the odds of detecting the turn signal turned on as a function of all of these factors.

A first important result of the survey to be highlighted is that only in 40% of the cases, the aspect of the turning indicators has been correctly identified (Table 1); this suggests that probably the characteristics of these devices, even if compliant with EU regulation and standards as described in part 1.2, must be improved and, in any case, that further research is needed in order to identify the factors that negatively affect their correct perception.

Moreover, within the limits of the proposed methodology, relationships seem to exist between the considered attributes and the odds of turn signal detection by motorcyclists. First of all, gender and urban context do not seem to have a statistically significant influence on the perception of turning indicators. Being a habitual cyclist seems to have a negative influence on the perception of the turn indicator, although slightly. Habitual car drivers have significantly higher chances of detecting the turning signal, probably due the usual conditions that drivers typically face on a roadway. The confidence of road users is a latent variable frequently related with hazard perception [45] and the results obtained in this study are in line with the literature [46]; in particular, confirming that confident and experienced road users (in this case motorcyclists who are also regular car drivers) show better hazard perception ability.

A tester’s age plays a big role in the signal detection, as younger users have higher detection rate compared to older people, and this is in line with the literature previously examined. Finally, the presence of a car occluding the view leads to higher detection rate of the blinker. This could be associated to the fact that an additional car behind the test vehicle is perceived as a higher risk situation, which increase the user’s awareness. The latter is probably the most interesting result of our analysis, as many accidents occur, in urban contexts, in such conditions.

While these are interesting preliminary results, considerably more research in this area is needed, because turn signal detection is a problem not only related to distracted drivers, but also to various environmental and driving conditions, and it should be further investigated in order to mitigate the risk of accidents. Multidimensional research approaches with the support of driving simulators should be adopted, in order to assess the ability to detect turning signals in potentially hazardous driving situations, exploring, in depth, the effects of the latent and explicit factors considered in this study.

Based on the videos contextually recorded from inside the vehicle during the simulations, we aim to design a new test campaign to also examine the reciprocal situation, that is the perception of motorcyclists overtaking the motorists stopped at the intersection.

As the main goal of the stakeholders involved in research and industrial development on road safety is to significantly reduce injuries, deaths and property damage, the preliminary results of this study show that we should take a serious collective look at the subject of turn signal detection and strive to proactively improve it so that all drivers appropriately perceive blinkers at all times.

## Figures and Tables

**Table 1:** Description of the study variables.

Variable	Description of Variable	Values	Count (*) (Proportion)
SIG	Correct detection	0 = no	164 (60%)
		1 = yes	108 (40%)
CYC	Bicycle Driver	0 = no	70 (51%)
		1 = yes	66 (49%)
CAR	Car Driver	0 = no	44 (32%)
		1 = yes	92 (68%)
GEN	Gender	0 = male	58 (43%)
		1 = female	78 (57%)
AGE	Age	0 = if ≥ 50	35 (26%)
		1 = if < 50	101 (74%)
OCCL	Occluding Car	0 = no	136 (50%)
		1 = yes	136 (50%)
URB	Urban	0 = no	136 (50%)
		1 = yes	136 (50%)

* Each individual in the sample, according to the experimental design, has viewed two videos.

**Table 2:** Estimated coefficients, estimated standard errors, *p*-values and Wald statistic for the model variables.

Variable	Coefficient	Std. Err.	Wald Statistic	*p*-Value
Intercept	−3.320	0.906	13.4	<0.001 *
CYC	−1.049	0.449	5.46	0.019 *
CAR	1.203	0.569	4.46	0.034 *
GEN	0.091	0.455	0.039	0.841
AGE	2.171	0.630	11.86	<0.001 *
OCCL	0.747	0.368	4.13	0.042 *
URB	0.209	0.386	0.294	0.587

* Statistically significant at 5% level.

**Table 3:** Effects of the estimated coefficients on the probability *p*(*x*).

Variable	Coefficient	Odds Ratio	Effect
CYC	−1.049	0.35	Negative
CAR	1.203	3.33	Positive
AGE (<50)	2.171	8.77	Positive
OCCL	0.747	2.11	Positive
